# Mitochondrial respiratory chain dysfunction in a patient with a heterozygous de novo *CTBP1* variant

**DOI:** 10.1002/jmd2.12326

**Published:** 2022-08-24

**Authors:** Wui‐Kwan Wong, Shanti Balasubramaniam, Rachel S. H. Wong, Nicole Graf, David R. Thorburn, Robert McFarland, Christopher Troedson

**Affiliations:** ^1^ TY Nelson Department of Neurology and Neurosurgery The Children's Hospital at Westmead Sydney New South Wales Australia; ^2^ Genetic Metabolic Disorders Service The Children's Hospital at Westmead Sydney New South Wales Australia; ^3^ Discipline of Genomic Medicine, Sydney Medical School University of Sydney Sydney New South Wales Australia; ^4^ Department of Histopathology The Children's Hospital at Westmead Sydney Australia; ^5^ Murdoch Children's Research Institute Melbourne Victoria Australia; ^6^ Department of Paediatrics University of Melbourne Melbourne Victoria Australia; ^7^ Victorian Clinical Genetics Services Melbourne Victoria Australia; ^8^ Wellcome Centre for Mitochondrial Research, Translational and Clinical Research Institute, Faculty of Medical Sciences Newcastle University Newcastle upon Tyne UK; ^9^ NHS Highly Specialised Service for Rare Mitochondrial Disorders Newcastle upon Tyne Hospitals NHS Foundation Trust Newcastle upon Tyne UK

**Keywords:** CTBP1, neurodevelopmental disorder, secondary mitochondrial dysfunction

## Abstract

The C‐terminal binding protein 1 (CTBP1) functions as a transcriptional corepressor in vertebrates and has been identified to have critical roles in nervous system growth and development. Pathogenic variants in the *CTBP1* gene has been shown to cause hypotonia, ataxia, developmental delay and tooth enamel defect syndrome (HADDTS). There have only been 16 cases reported to date with heterozygous, pathogenic variants in *CTBP1* manifesting with a neurodevelopmental phenotype. We report a further case of a pathogenic, heterozygous, de novo variant in *CTBP1* identified by whole exome sequencing in a female with the typical phenotype of global developmental delay, hypotonia, cerebellar dysfunction and failure to thrive. Additionally, muscle biopsy demonstrates evidence of a respiratory chain defect, only previously reported once in the literature. This supports the role of CTBP1 in maintenance of normal mitochondrial activity and highlights the importance of considering secondary mitochondrial dysfunction in genes not directly involved in the mitochondrial respiratory chain.

**Synopsis**

Pathogenic variants in the *CTBP1* gene may be associated with secondary mitochondrial dysfunction.

## 1 INTRODUCTION

In vertebrates, the C‐terminal binding protein (CTBP) family is made up of CTBP1 and CTBP2, which function as transcriptional corepressors mediated by a high‐affinity protein‐binding interface, known as the PXDLS‐binding cleft.^1^, ^2^ CTBP1 is ubiquitously expressed in all tissues in vertebrates and plays critical roles in gene regulation during human development, with its importance for neurodevelopment being mediated via regulation of genes involved in neuron survival, growth, membrane excitability, synaptic transmission and plasticity.^2^ Pathogenic variants in the *CTBP1* gene have been previously reported to cause an early onset neurodevelopmental phenotype with associated decreased mitochondrial respiratory chain activities in a single case report.^1^, ^3^, ^4^, ^5^, ^6^, ^7^, ^8^

A variety of neurodevelopmental abnormalities are common manifestations of mitochondrial disorders and an increasing number of well‐known genes associated with neurodevelopmental disorders show evidence of impaired mitochondrial function.^9^, ^10^, ^11^ We present the second reported case of mitochondrial dysfunction in a 6‐year‐old girl with previously reported pathogenic heterozygous de novo missense variant in the *CTBP1* gene. Our patient presented with global developmental delay, central hypotonia, cerebellar dysfunction and poor weight gain with muscle biopsy demonstrating evidence of a respiratory chain defect primarily affecting complex IV.

## 2 CASE REPORT

The proband is a now 6‐year‐old female who initially presented to the paediatric neurology clinic at 20‐month‐old with failure to thrive, global developmental delay and central hypotonia from 8 months of age.

She is the second child to non‐consanguineous parents of Caucasian descent (maternal family from Scotland; paternal family from England and Australia). There was no family history of neurodevelopmental disorders. Her antenatal and perinatal history was unremarkable. Birthweight was 3.182 kg (33rd centile) and head circumference at birth was 34.4 cm (42nd centile). However, her weight subsequently dropped to be persistently below the 3rd centile from 8 months of age despite adequate intake and no evidence of malabsorption. At 5‐year‐old, the patient's weight was 15.5 kg (<3rd centile), height 100.5 cm (<1st centile) and head circumference 48.5 cm (5th centile).

The parents reported normal development until 6 months of age when she was able to sit independently and was babbling. Thereafter, she has had slow developmental progress in all domains without regression. At 5‐year‐old, she was able to stand with support but not independently. She was right‐hand dominant, able to hold a pen and form a rough linear scribble and navigate tablet applications appropriately. She was able to babble but had no spoken language and was able to understand simple, one‐step commands. She was not yet toilet trained and unable to dress or undress herself. She was noted to have frequent mild viral infections, including two episodes of developmental regression in association with an intercurrent viral illness.

On serial examinations over several years, she was profoundly hypotonic. Deep tendon reflexes were difficult to elicit. Eye movements were abnormal with saccadic pursuit, slow saccades and end gaze nystagmus. She had ataxia and weakness of all four limbs. There was no evidence of facial weakness or bulbar dysfunction. Her spine was straight. There was mild discoloration on dental examination with generalised mild hypomineralisation.

**FIGURE 1:**
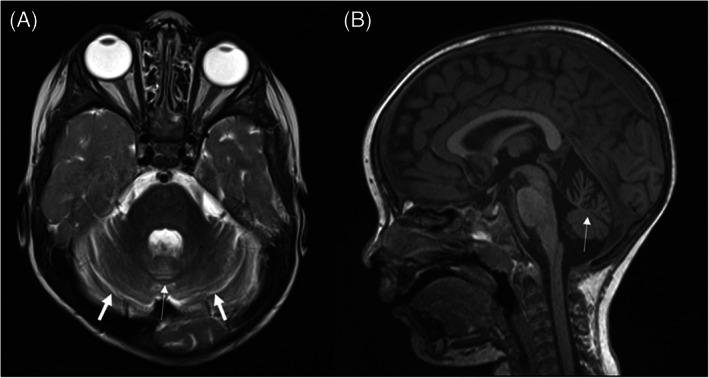
MRI brain performed at 2 years old showing cerebellar atrophy affecting the vermis (thin arrow) more than the cerebellar hemispheres (thick arrows)

Nerve conduction studies and electromyogram at 23‐month‐old were normal. MRI head at 2‐year‐old showed global cerebellar atrophy affecting the vermis more than the cerebellar hemispheres (Figure 1). Electroencephalogram was normal in the awake state. Repeat urine metabolic screens showed generalised aminoaciduria with more significant increases in cystine, lysine, arginine and ornithine to creatinine ratios suggesting heterozygous cystinuria (detailed results of urine amino acid analysis are provided in Table S1). Urine organic acid analysis performed at 4 years of age showed moderately increased lactate, and was subsequently slightly increased when repeated 6 months later. Further analysis of urine organic acids at 5 years was normal. Plasma lactate, amino acids, transferrin isoforms, plasma very long chain fatty acids, acylcarnitine profile, oligosaccharide screen, alpha‐galactosidase activity and lyso‐sphingomyelin‐509 levels were normal on initial testing at 3 years of age. A single blood gas analysis showed mild acidosis (pH 7.34) with a normal lactate (0.9 mmol/L). Cerebrospinal fluid (CSF) cell counts, glucose, protein, lactate, 5‐methyltetrahydrofolate, amino acids and neurotransmitters were normal.

**FIGURE 2:**
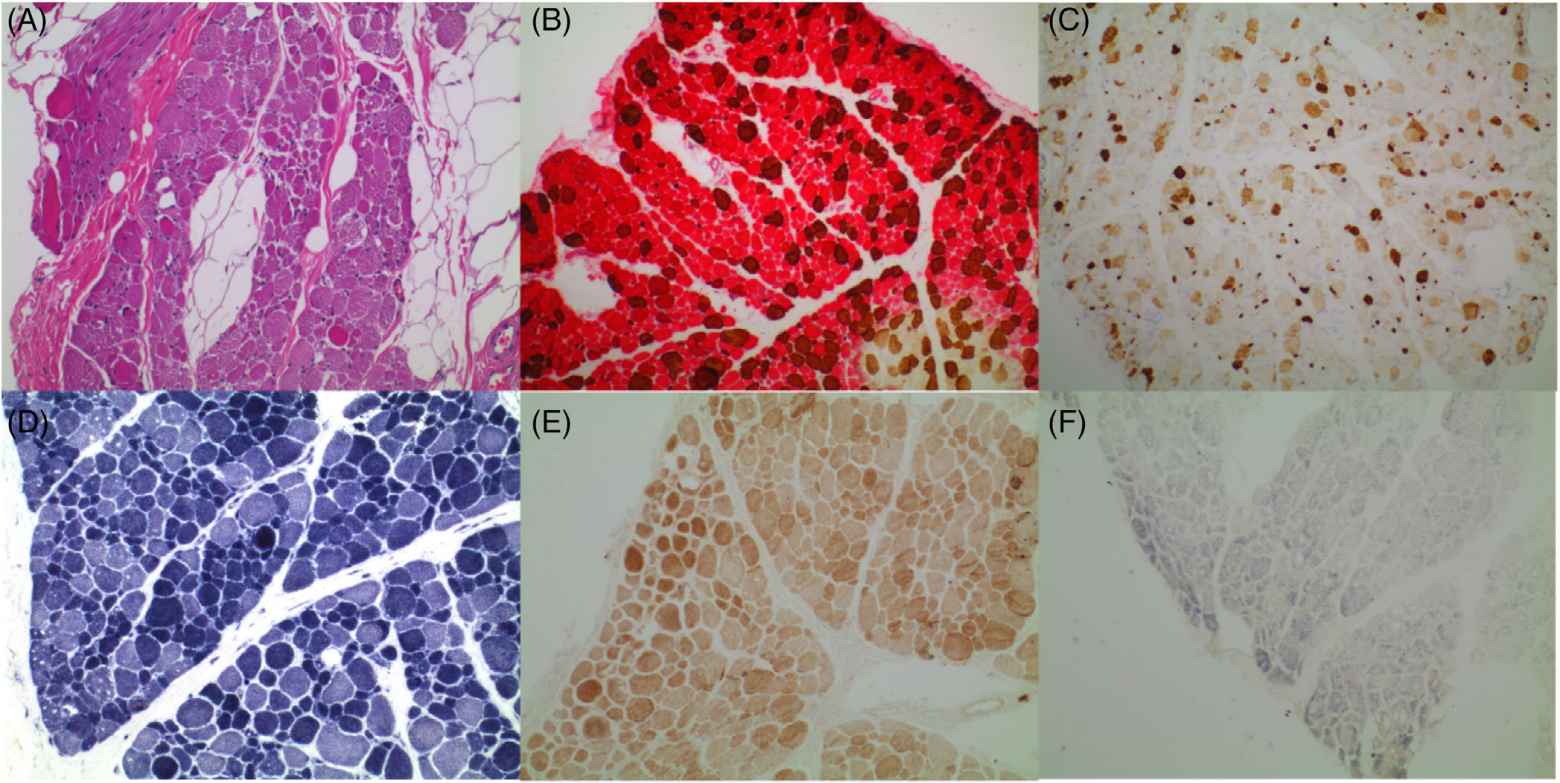
(A) Haematoxylin and eosin (H&E) stained formalin‐fixed paraffin‐embedded (FFPE) section (×100 magnification) demonstrating infiltration of skeletal muscle by adipose tissue, with fibre size variation and focal endomysial fibrosis. (B) Dual muscle myosin stain performed on frozen section of slow muscle myosin (red) and fast muscle myosin (brown) confirms predominance of type 1 fibres. (C) Neonatal myosin (×10 magnification) performed on frozen section demonstrates abnormal expression for neonatal myosin in a proportion of the muscle fibres. (D) NADH enzyme stain. (E) COX‐SDH enzyme stain. (F) SDH enzyme stain. Oxidative enzyme stains (NADH, COX‐SDH and SDH) demonstrate the variation in fibre size and relative type 1 predominance. There is no evidence of COX deficiency with retained staining, however the SDH stains were considered technically suboptimal. COX, cytochrome c oxidase; NADH, nicotinamide adenine dinucleotide; SDH, succinate dehydrogenase

Microscopic muscle biopsy analysis of skeletal muscle (left vastus lateralis) was abnormal with non‐specific myopathic changes including marked fibre size variation with plentiful atrophic fibres, marked type 1 fibre predominance, a mild increase in internalised nuclei and adipose tissue infiltration (Figure 2A,B). Abnormal expression for neonatal myosin was also present in a proportion of the muscle fibres (Figure 2C). Oxidative enzymes stains, including nicotinamide adenine dinucleotide‐tetrazolium reductase (NADH‐TR) and cytochrome c oxidase (COX), showed a normal pattern with a substantial reduction (but not total absence) of staining for succinate dehydrogenase (SDH) (Figure 2D–F). Respiratory chain enzymology primarily showed decreased complex IV activity (6% of control), with complex I activity (23% of control) and complex II + III activity (15% of control) also decreased (Table 1). This was diagnostic of a respiratory chain defect affecting primarily complex IV.

**TABLE 1:** Respiratory chain enzyme analysis in patient skeletal muscle

	Activity		Reference range	% Activity	% CS ratio	% CII ratio
Complex I (NADH‐coenzyme Q1 oxidoreductase)	9 nmol/min/mg	L	(19–72)	**23**	**26**	35
Complex II (succinate‐coenzyme Q1 oxidoreductase)	30 nmol/min/mg		(26–63)	66	75	
Complex II + III (succinate cytochrome c reductase)	7 nmol/min/mg	L	(30–76)	**15**	**17**	**23**
Complex III (decylbenzylquinol‐cytochrome c oxidoreductase)	14.2 nmol/min/mg		(13–51)	49	53	72
Complex IV (cytochrome c oxidase)	0.40 nmol/min/mg	L	(3.3–9.1)	**6**	**7**	**9**
Citrate synthase	113 nmol/min/mg		(85–179)	88		

*Note*: Enzyme activities are shown as absolute values and as % residual activity relative to protein (% activity), citrate synthase (% CS ratio), and complex II (% CII ratio). % values <30 are shown in bold and represent major (<20) or minor (<30) criteria in the Bernier Diagnostic Scheme.^16^

Trio whole exome sequencing at 3.5‐year‐old identified a previously reported de novo, pathogenic heterozygous missense variant in the *CTBP1* gene (NM_001012614.1:c.991C>T, p.[Arg331Trp]), consistent with the patient's clinical presentation. Of note, given the urine metabolic testing results, no significant variants were identified in *SLC7A9* and *SLC3A1*, the two genes responsible for cystinuria.

This patient was trialled on a metabolic cocktail of coenzyme Q10 150 mg daily (previously started at 2.5 years old when first clinically suspected to have a mitochondrial disorder) with the addition of ascorbic acid 50 mg daily, riboflavin 50 mg daily and vitamin E 200 IU daily at 4 years old after the results of whole exome sequencing returned identifying the variant in *CTBP1*. Upon subsequent review, the family reported less frequent infections, tolerated two surgeries (insertion of gastrostomy and major orthopaedic surgery) and chose to continue medications.

## 3 DISCUSSION

Hypotonia, ataxia, developmental delay and tooth enamel defect syndrome (HADDTS; MIM: 617915) secondary to a recurrent de novo missense mutation in *CTBP1* [NM_001012614.1:c.991C>T, p.(Arg331Trp)] was first reported by Beck et al.^3^ They described four unrelated individuals with a neurodevelopmental phenotype consisting of early onset developmental delay, intellectual impairment, hypotonia, cerebellar dysfunction, poor weight gain, tooth enamel defects and variable changes on muscle biopsy.^3^ Since then, a further 11 individuals have been reported in the literature with the same variant in *CTBP1*, and one individual with a de novo frameshift pathogenic variant in *CTBP1* (c.1315_1316delCA, p.Gln439ValfsTer84) with similar phenotypes (Table 2).^1^, ^4^, ^5^, ^6^, ^7^ Of note, only one individual had a muscle biopsy demonstrating decreased mitochondrial respiratory enzymes activities in complex I and IV with additional patchy loss of COX activity and clumped SDH reactivity.^7^ A further case reported persistently elevated serum lactate levels with muscle biopsy demonstrating features of muscular dystrophy but respiratory chain enzyme analysis was not reported.^8^ As yet, generalised aminoaciduria observed in our patient has not been reported in this disorder. We speculate that the underlying pathogenesis could be attributed to proximal renal tubulopathy, which is the most frequent renal manifestation of mitochondrial dysfunction.^12^ Our case contributes to the evidence that secondary mitochondrial dysfunction may contribute to the pathogenesis in the *CTBP1*‐related neurodevelopmental disorder.

**TABLE 2:** Characteristics of individuals with pathogenic variants in *CTBP1*

Reference	Age (years)	Sex	*CTBP1* variant	Development delay	Growth failure	Dental abnormality	Cerebellar features	Dysmorphic features	Neuromuscular features	MRI head	Muscle biopsy
This patient	6	F	c.991C>T	+ global	+	Reduced mineralisation with discoloration	+	−	Hypotonia, limb weakness, reduced DTRs	Cerebellar atrophy	Fibre size variation, atrophic fibres, increase in internalised nuclei and fatty infiltration Decreased complex IV, I and II + III activity
^3^	10	M	c.991C>T	+ global	+	Enamel defect	+	−	Hypotonia, weakness	Cerebellar volume loss	Perivascular inflammation
	23	M	c.991C>T	+ global	+	Soft enamel with discoloration	+	Frontal bossing, deep set eyes	Hypotonia, weakness, increased lower limb reflexes	Cerebellar atrophy	Varied muscle fibre size, endomysial connective tissue
	9	F	c.991C>T	+ motor	+	Wide‐spaced incisors, discoloration of primary incisors	+	Retrognathia, high arched palate	Hypotonia, weakness, absent DTRs, hyperflexibility	Superior vermis volume loss	Inflammatory infiltrates around perimysial blood vessels and occasional degenerating or necrotic fibres
	12 (deceased)	F	c.991C>T	+ global	+	Enamel hypoplasia	+	−	Hypotonia	Normal	Dystrophic changes with marked variability in fibre size and shape with both atrophic and hypertrophied fibres, scattered pyknotic nuclear clumps, adipose tissue
^7^	16 (deceased)	F	c.991C>T	+ global	NR	−	−	−	Hypotonia, generalised weakness	Mild cerebellar and brainstem atrophy	Fibre size variation, occasional internal nuclei, denervation atrophy, accumulation of fibroadipose connective tissue, patchy lipid accumulation. Decreased complex I and IV activities
^4^	8	F	c.991C>T	+ global	NR	Enamel defects	+	NR	Hypotonia EMG: myogenic	NR	Atrophic and hypertrophic fibres; intermyofibrillar network disruption; disruption of sarcomeres and absence of mitochondria
^1^	20	M	c.991C>T	+ global	NR	Enamel dysplasia	+	NR	Hypotonia EMG: myopathic	NR	NP
	22	F	c.991C>T	+ global	NR	Protuberant malpositioned teeth, widely spaced incisors, discoloration of roots	+	NR	Hypotonia, limb girdle and bulbar weakness, decreased muscle bulk, absent DTRs, contractures of hips, knees and elbows EMG: bilateral ulnar neuropathies	Progressive cerebellar loss. Mild enlargement of cerebral sulci	NP
	6	M	c.991C>T	+ global	NR	Enamel defects	+	NR	Hypotonia, muscle weakness	Normal	NP
^1^	6	M	c.991C>T	+ global	NR	Enamel dysplasia	−	NR	Hypotonia, absent DTRs, mild hyperextensibility at major joints, positive Babinski sign EMG: diffuse myopathy	Cerebellar atrophy (mainly superior)	NP
	10	M	c.991C>T	+ global	NR	Unspecified abnormalities, multiple cavities	+	NR	Axial hypotonia, increased ankle tone, absent DTRs at knees, weakness in hands and lower limbs	Superior cerebellar vermis atrophy and mild Dandy Walker cyst	NP
	5	M	c.991C>T	+ global	NR	NR	+	NR	Hypotonia	Underdeveloped cerebellum	NP
	11	M	c.991C>T	+ global	NR	Enamel discoloration, crowded dentition	+	NR	Hypotonia	Cerebellar and pons atrophy	NP
^8^	7	M	c.991C>T	+ global	+	Enamel defects, wide spaced upper incisors	−	Long face (myopathic)	Generalised muscle wasting, neck muscle weakness, truncal hypotonia, absent DTRs, elbow and knee joint contractures, hyperlaxity in the distal interphalangeal joints Elevated CK EMG: myopathic	Prominent cerebellar foliae	Features of muscular dystrophy
^5^	16	M	c.991C>T	+ global	NR	Teeth discoloration	+	NR	Hypotonia, proximal muscle weakness, absent DTRs Mildly elevated CK EMG: myopathic	Cerebellar atrophy	Dystrophic pattern
^6^	26	M	c.1315‐1316delCA	+ global	−	Enamel defect, teeth deformation, brown discoloration of roots	+	−	Mild joint hyperextensibility	NP	NP

Abbreviations: EMG, electromyogram; DTRs, deep tendon reflexes; NP, not performed; NR, not recorded; +, present; −, absent.

Mitochondrial oxidative phosphorylation (OXPHOS) comprises four complexes (the respiratory chain) that play a primary role in energy production via generation of an electrochemical gradient across the inner mitochondrial membrane. OXPHOS complex V uses this gradient to generate energy in the form of adenosine triphosphate (ATP).^10^, ^11^ Primary mitochondrial disorders result from genetic variants in either mitochondrial DNA or nuclear DNA that encode OXPHOS proteins or impact the production of machinery needed for OXPHOS to perform optimally.^10^ Essentially any organ system can be involved in mitochondrial disease, with neurodevelopmental abnormalities being extremely common and include hypotonia, weakness, developmental delay and failure to thrive; all features seen in this case. Additionally, secondary mitochondrial dysfunction has been increasingly described in a number of neurodevelopmental disorders with known causative genes that do not fall into the previous categories, including fragile X syndrome, Angelman syndrome, tuberous sclerosis and Rett syndrome.^11^ While there are rare mitochondrial disorders for which specific therapies are indicated, regular caloric intake and empiric “mitochondrial cocktails” are often used as part of management and may include B vitamins, vitamin E, vitamin C and antioxidants including coenzyme Q10.^9^, ^10^ Specific to our patient, riboflavin is a flavoprotein precursor and a key building block in complex I and II, and has been used in treating mitochondrial diseases affecting these complexes.^13^, ^14^ Furthermore, coenzyme Q10, vitamin C and vitamin E are antioxidants, that is, compounds that scavenge free radicals by accepting unpaired electrons and becoming reduced. They are used in mitochondrial disease to minimise cellular oxidative stress damage and hence we chose this choice of therapy in our patient. Whilst these “mitochondrial cocktails” are relatively benign, the evidence to support the effectiveness of these interventions is limited and clinicians largely depend upon observations of clinical benefit to determine whether to continue, such as occurred in this patient.^9^

**FIGURE 3:**
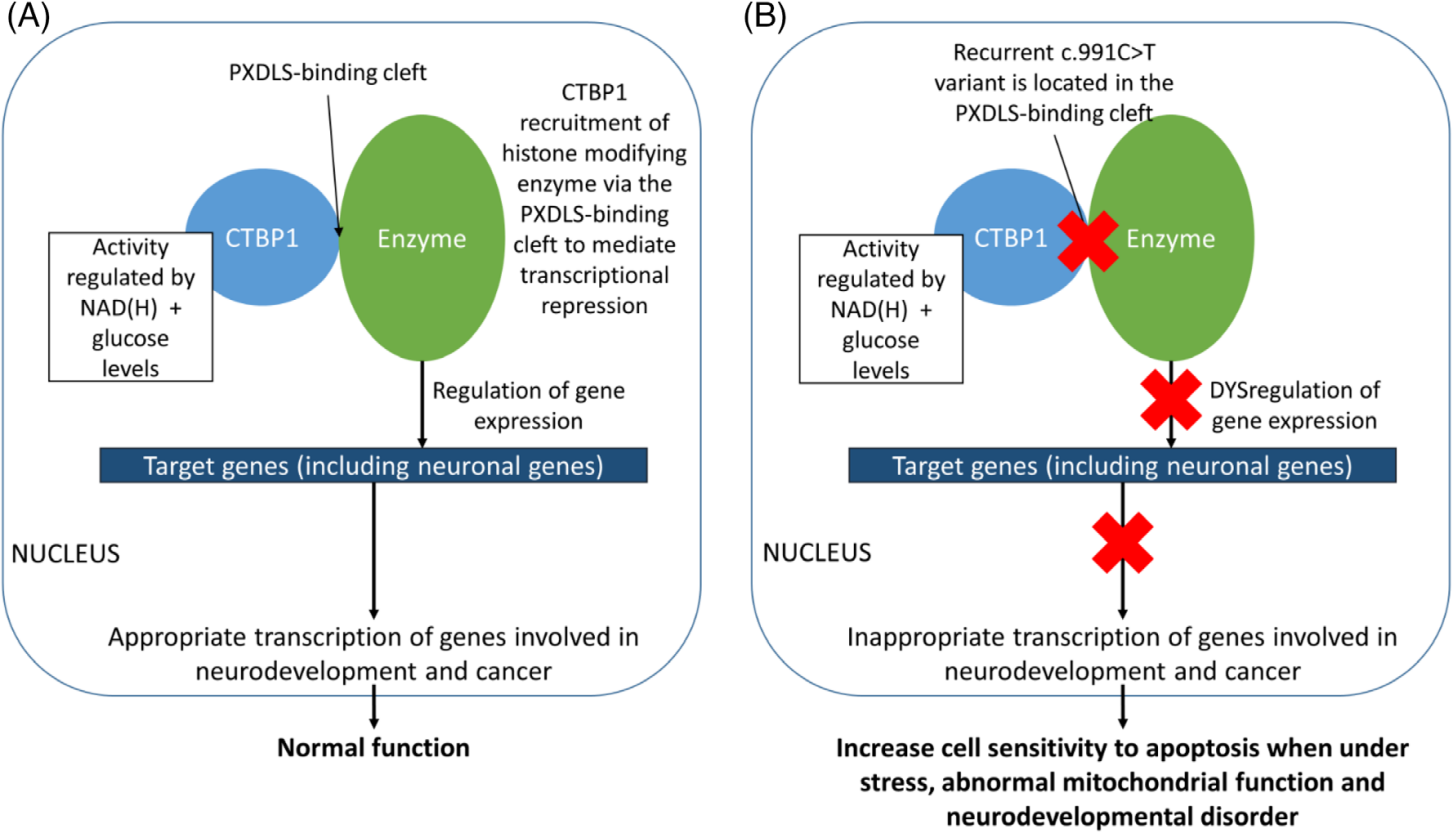
Diagram demonstrating the current understanding of the CTBP1 protein in human neurodevelopment (A) and the proposed effect of dysfunction of the protein in the recurrent c.991C>T variant of the *CTBP1* gene (B).^1^, ^2^ CTBP1, C‐terminal binding protein 1

As previously mentioned, CTBP1 is essential for normal human neurodevelopment and maintains transcriptional repression by targeting chromatin‐modifying enzymes to gene promotor regions and interacting with DNA‐bound repressors via the PDXLS binding cleft (Figure 3).^2^ Although CTBP1 does not localise to the mitochondria, it acts as a metabolic sensor by differentially binding to NADH to control downstream gene transcription with intracellular NADH levels differentially regulating transcriptional activity.^2^, ^15^ Knockout of *CTBP1* in mouse models demonstrated increased sensitivity to apoptosis when exposed to low glucose levels, abnormal mitochondrial morphology and abnormal cellular ATP level, oxygen consumption and mitochondrial membrane potential.^15^ CTBP1 regulates the pro‐apoptotic Bcl‐2 associated X protein (*Bax*) transcription, associating with the *Bax* promotor region during normal glucose states and disassociating in response to glucose deprivation.^15^ Furthermore, fibroblasts from patients with the c.991C>T variant were found to have more cell death than healthy controls when deprived of glucose, with evidence of elevated transcript and protein levels of the pro‐apoptotic gene *Noxa*.^1^ The recurrent c.991C>T variant has been demonstrated to be located in the PDXLS binding cleft.^1^ These functional studies show the importance of CTBP1 in maintaining normal mitochondrial activity and suggest that CTBP1 dysfunction may result in dysregulated apoptosis in the nervous system contributing to the well‐described neurodevelopmental phenotype (Figure 3).

The identification of a pathogenic, heterozygous, de novo variant in *CTBP1* by whole exome sequencing in an individual with a neurodevelopmental phenotype and muscle biopsy suggestive of mitochondrial disease is a prime example of the importance of closely analysing de novo variants for pathogenicity and additionally, considering secondary mitochondrial dysfunction in genes not directly involved in OXPHOS. This has implications for clinical management, including avoidance of potentially “mitotoxic” medications, precautions during illnesses and anaesthetics, and considering trialling supplements with close observation for a beneficial effect.

## AUTHOR CONTRIBUTIONS

Wui‐Kwan Wong and Shanti Balasubramaniam drafted the outline of the manuscript. Shanti Balasubramaniam, Christopher Troedson and Rachel SH Wong were involved in the clinical management of the patient. David R Thorburn performed and interpreted mitochondrial respiratory chain enzyme activities on this patient's tissue samples. Robert McFarland advised on the diagnostic work‐up. Nicole Graf performed and interpreted muscle biopsy analysis. All authors have read/critically revised the manuscript.

## FUNDING INFORMATION

David R Thorburn is supported by a National Health and Medical Research Council Principal Research Fellowship GNT1155244.

## CONFLICT OF INTEREST

Wui‐Kwan Wong, Shanti Balasubramaniam, Rachel SH Wong, Nicole Graf, David R Thorburn, Robert McFarland, and Christopher Troedson declare that they have no conflict of interest.

## ETHICS APPROVAL

Ethics approval was obtained from the local human research ethics committee (CCR2021/21).

## INFORMED CONSENT

Written informed consent was obtained from the patient's family for being included in this case report.

## PATIENT CONSENT

Written consent was gained from the patient's family.

## ANIMAL RIGHTS

This article does not contain any studies with human or animal subjects performed by any of the authors outside the realms of clinical care.

## Supporting information


**TABLE S1** Patient's urine amino acid analysis in μmol/mmol by tandem mass spectrometry quantification collected over 12 months

## Data Availability

All data specific to the case report can be accessed via electronic medical records at The Children's Hospital at Westmead, Sydney, NSW, Australia. All other data used in the manuscript has been referenced in the body and reference list. No publicly archived datasets were used during the study.
