# Supplementary material for: Mitochondrial respiratory chain dysfunction in a patient with a heterozygous de novo CTBP1 variant
Source: JIMD Rep. 2022 Aug 24;63(6):546–54. doi: 10.1002/jmd2.12326 (PMC9626656; doi:10.1002/jmd2.12326)
Supplement: Supplementary file 1 — TABLE S1 Patient's urine amino acid analysis in μmol/mmol by tandem mass spectrometry quantification collected over 12 months [file JMD2-63-546-s001.docx]

|  | **At 4-years** | **At 4.5-year** | **At 5-years** | **Reference range** |
| --- | --- | --- | --- | --- |
| Alanine/creatinine | 223 | 192 | 276 | 13-183 |
| Arginine/creatinine | 50 | 30 | 57 | 1-17 |
| B-AIBA/creatinine | 15 | 74 | 32 | 1-168 |
| Citrulline/creatinine | 9 | 9 | 17 | 0-7 |
| Cystine/creatinine | 333 | 224 | 271 | 1-29 |
| Glutamine/creatinine | 263 | 5197 | 430 | 30-299 |
| Glycine/creatinine | 823 | 797 | 901 | 63-812 |
| Homocystine/creatinine | 0 | 1 | 0 | 0-3 |
| Leucine/creatinine | 21 | 282 | 111 | 2-80 |
| Lycine/creatinine | 468 | 240 | 717 | 5-74 |
| Methionine/creatinine | 10 | 8 | 10 | 1-9 |
| Ornithine/creatinine | 73 | 57 | 85 | 1-14 |
| Phenylalanine/creatinine | 23 | 28 | 24 | 3-33 |
| Phosphoethanolamine/creatinine | 3 | 2 | 1 | 0-19 |
| Pipecolic acid/creatinine | 5 | 7 | 0 | 0-29 |
| Proline/creatinine | 5 | 2 | 35 | 1-15 |
| Serine/creatinine | 270 | 315 | 287 | 24-202 |
| Threonine/creatinine | 113 | 107 | 148 | 0-82 |
| Tyrosine/creatinine | 78 | 68 | 73 | 11-74 |
| Valine/creatinine | 29 | 33 | 44 | 3-26 |

**Supplementary Table 1:** Patient’s urine amino acid analysis in umol/mmol by tandem mass spectrometry quantification collected over 12 months
